# Numerical Study on Local Entropy Production Mechanism of a Contra-Rotating Fan

**DOI:** 10.3390/e25091293

**Published:** 2023-09-03

**Authors:** Xingyu Jia, Xi Zhang

**Affiliations:** School of Mechanical Electronic & Information Engineering, China University of Mining & Technology-Beijing, Ding No. 11 Xueyuan Road, Haidian District, Beijing 100083, China; jiaxy_cumtb@163.com

**Keywords:** contra-rotating fan, local entropy production, tip leakage flow, shear strain rate, optimal design of radial load

## Abstract

Contra-rotating fans (CRFs) have garnered significant attention due to their higher power-to-weight ratio compared to traditional fans; however, limited focus has been given to the localization and development of local aerodynamic losses. Furthermore, there is a need for further research on the impact of load distribution along the radius on local entropy production. Therefore, this study aims to investigate a contra-rotating fan as the research subject. An optimal design for load distribution along the radius is achieved by constructing a surrogate model in combination with a genetic algorithm. The effectiveness of this design has been verified through experimentation using a specific test device. In this study, a local entropy production rate (EPR) model adapted to the shear stress transport-detached eddy simulation (SST-DES) technique is constructed to evaluate the loss distribution of the contra-rotating fan. This paper primarily focuses on comparing and analyzing the blade profile and overall performance of the CRFs before and after optimization. The EPR contribution of each interval along the radius is compared to the corresponding blade channel to identify the approximate range of high-EPR regions. Furthermore, an investigation is conducted to examine the distribution of EPR along the streamwise direction in these high-EPR regions. After that, by comparing the development of the flow structure near a stall before and after optimization, combined with the analysis of the EPR contours, the EPR mechanism of this CRF is revealed.

## 1. Introduction

In light of the growing global resource challenges, there is a pressing need to enhance the efficiency of energy-intensive equipment. Specifically, when it comes to ventilators, it is crucial to minimize aerodynamic loss and broaden their high-efficiency operating range. By doing so, it cannot only reduce energy consumption and save resources but also enhance the overall stability of the system.

The operation of an axial flow ventilator involves the periodic interaction between the vortex systems generated by the blade tip, root, and trailing edge with the boundary layer developed by the blade surface and annulus. This interaction leads to aerodynamic loss [1].

In comparison to the traditional rotor–stator stage, the contra-rotating stage can recover the rotational kinetic energy from upstream by anti-rotating its rear rotor (RR). This allows for a higher power-to-weight ratio and flow capacity in a more compact configuration; however, compared to the traditional stage, the cancellation of the guide vanes between the front rotor (FR) and RR in some contra-rotating stages leads to increased periodic interactions between the vortex systems and the developed boundary layer. This, in turn, results in higher aerodynamic losses in the rear stage compared to the front stage.

With respect to the assessment of the overall aerodynamic loss of the axial flow blades, in many investigations [2,3,4], this loss can be conveniently evaluated by measuring the pressure parameters at the entrance and exit of the blade passage and calculating the relative total pressure loss coefficient; however, the relative total pressure loss coefficient is a cumulative quantity, so this coefficient cannot express the local contribution to the loss very intuitively.

According to the second law of thermodynamics, entropy production is directly related to the loss in an irreversible process [5], which provides an idea for describing the local loss. According to the entropy transport equation, entropy production in turbomachines consists of viscous loss due to the shear strain rate and heat transfer due to the temperature gradient [6,7]. In combination with the computational fluid dynamics (CFD) technique, Kock and Herwig [8] proposed an entropy production rate model suitable for the flow case solved using the RANS equations, which is currently widely used [9,10,11,12,13].

At present, most investigations on the aerodynamic losses of contra-rotating ventilators have been calculated based on the Reynolds-averaged Navier–Stokes equations (RANS) model framework. Based on the RANS model, Yang and Shan [14] compared the loss generation from military and civilian CRFs at different speed ratios. The aerodynamic losses of the two fans showed the same tendency with the variation in their rotational speeds; as the rotational speed of the RR increases, the aerodynamic loss of the rear stage increases rapidly. The rear stage was the main contributor to the aerodynamic loss generation. Joly et al. [15] established an optimal design platform for contra-rotating fan blades using a RANS model. After calculating the radial distribution of the entropy production for each rotor, they concluded that the entropy production of the RR was higher than that of the FR. Based on the unsteady-RANS (URANS) model, Luan et al. [16] compared the relative entropy coefficients of a CRF along the radius for five different axial spacings. They concluded that, as the axial spacing increased, the unsteady effect of both rotors weakened, resulting in less aerodynamic loss generation. Based on the RANS model, Bandopadhyay and Mistry [17] compared the total pressure loss of a CRF under four load allocations. They found that, as the load allocation of the front stage increased, the low-energy fluid generated by the FR increased, and the total pressure loss between the rotors increased.

To accurately locate and evaluate entropy production, it is necessary to improve the resolution of flow structures through high-resolution simulations such as direct numerical simulation (DNS) or large eddy simulation (LES); however, conducting these simulations for large-scale engineering problems requires significant computing resources. The detached eddy simulation (DES) method [18] in scale-resolving simulations, which combines the advantages of the RANS and LES methods, can help reduce the consumption of computing resources. While this method has been successfully used to locate aerodynamic loss in blades [19,20,21], it has rarely been applied to the study of aerodynamic loss in CRF blade passages.

Moreover, previous studies on the aerodynamic loss of CRFs have primarily focused on investigating the cumulative spatial loss along the flow direction; however, there is a need for further research on local entropy production in CRFs. Additionally, limited studies have been conducted on the relationship between the radial load distribution of the CRF blades and the induced local loss. Therefore, this study aims to optimize the CRF by considering the radial load coefficient and axial spacing as optimization variables. The SST-DES turbulence model is utilized to analyze the flow field before and after the CRF blade optimization; furthermore, a local entropy production model suitable for the SST-DES model is developed to quantitatively analyze the local entropy generation in the blade channel.

The structure of this paper is organized as follows: Section 2 introduces the blade design and optimization methods. Section 3 discusses the numerical methods and experimental verification results. Section 4 presents the local entropy production model and its expression under the SST-DES model. Section 5 presents the results of the optimization and transient analyses. The nomenclature used in this paper is listed in Abbreviations.

## 2. Design and Optimization

### 2.1. Blade Design Method

**Figure 1:**
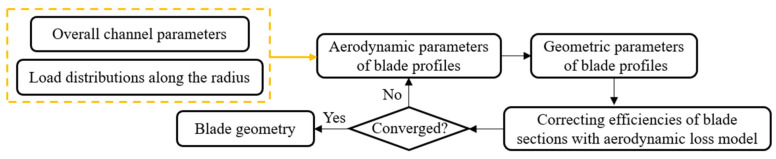
Design process of the CRF blade.

CRF blades studied in this article are designed and generated based on the S1/S2 stream surface [22] and cascade aerodynamics theory. There are 13 blades in the FR and 11 blades in the RR. The FR and RR are counter-rotating at 2950 rpm, respectively. The diameter of the blade channel is 602 mm with a hub/tip ratio of 0.598. The tip clearance is 1.5 mm. This CRF is designed to operate at a mass flow rate of 9.14 kg/s and a total pressure of 4860 Pa. The total pressure rise in the FR is equal to RR, which is 2430 Pa, respectively. The flowchart of the design process is shown in Figure 1.

**Figure 2:**
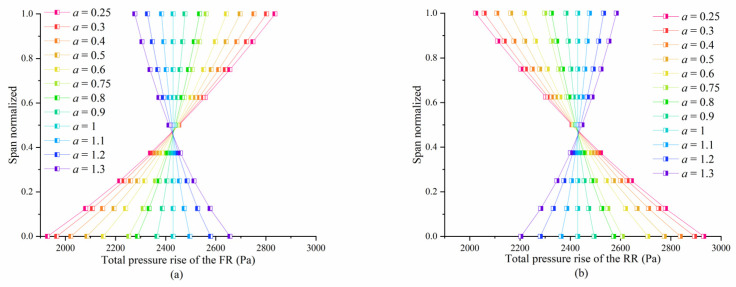
Results of the fine-tuning (total pressure rise) along the radius in the (**a**) FR and (**b**) RR.

To investigate the effect of the radial load distribution on the local entropy generation in the CRF blade passages, the key point is to establish a series of CRF blades by fine-tuning the total pressure rise along the radius in the S2 design phase. Another factor that must be considered is the axial spacing between the FR and RR. This is because the axial spacing can control the extent of interaction between the two rotors [23], which significantly influences the distribution of aerodynamic losses. The specific blade design method is fully described in [24], and only the description of the theoretical radial load distribution result is summarized here. The fine-tuning of the front and corresponding rear stages is complementary to maintaining the overall performance. That is, at a specific blade span, if the blade element of the FR is tuned by increasing its total pressure rise, the corresponding element of the RR would be tuned by decreasing it by the same value. The extent of the fine-tuning can be quantified using the parameter *a*, as shown in Figure 2.

### 2.2. Optimization

**Figure 3:**
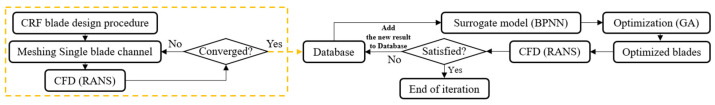
Flowchart of the CRF blade optimization.

The flowchart of the optimization process is shown in Figure 3. As mentioned in the introduction, this paper takes radial load coefficient and axial spacing as optimization variables, represented by ‘*a*’ and ‘*x*’ here, respectively.

**Figure 4:**
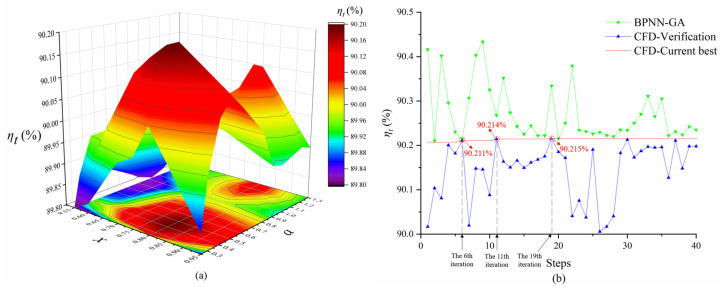
(**a**) Initial curved surface of the database, (**b**) iterations of the optimization.

Owing to the limitations of the aerodynamic performance and geometric parameters, the plane boundary of the variables is calculated and ultimately determined as the horizontal plane shown in Figure 4a. These details are also fully described in [24]. A total of 72 sets of blades are created by a sequential combination of 12 different ‘*a*’ with 6 different ‘*x*’. Next, the single-passage flow field is calculated by solving the RANS equations combined with the SST k-ω [25] turbulence model in the ANSYS CFX solver. Subsequently, after collecting *η_t_* (total pressure rise efficiency), the initial curved surface with its projection representing the database is shown in Figure 4a.

Then, during optimization, to renew the relationship between the optimization variables and *η_t_* after adding a new sample to the database, each iteration requires retraining a surrogate model established by a backpropagation neural network based on the steepest descent techniques [26]. The database is randomly assigned to the training and testing sets in a 7:3 ratio. The learning rate for each iteration is set at 0.5 to achieve a balance between convergence efficiency and accuracy.

The artificial neurons of this backpropagation neural network are connected in five layers with three hidden layers containing six neurons, respectively. Two inputs are the ‘*a*’ and ‘*x*’ neurons, and the output is *η_t_*. The reason why *η_t_* is chosen as the single output in this study is due to the complexity that determines efficiency at the design condition: although the optimal incidence angle has been corrected for different thicknesses and cambers [27], it needs to achieve minimum aerodynamic losses under the design conditions in the current design process to improve the stall margin and extend the effective operating range. However, mutual interference between the contra-rotational rotor blades further increases the difficulty of predicting the optimal incidence angle closely related to cascade loss. Consequently, this study considers *η_t_* under the design conditions as a fitness function and searches for a better candidate using a genetic algorithm [28]. Genetic algorithms are widely employed for turbomachine optimization [29], and this is a stochastic optimization algorithm [30]. Even if the function has discontinuities and noise, it may find an acceptable optimal solution [31]. The main settings of the genetic algorithm are listed in Table 1. The complete iteration of the optimization is shown in Figure 4b.

Finally, it has been confirmed that a satisfied maximum (90.22%) is met at the 19th iteration after two improvements (at the 6th and 11th iterations). Compared with the efficiency of the baseline model, which is 90.02% under the design conditions, the improvement is approximately 0.22%. The optimized variables corresponding to the satisfied maximum are 0.4025 for the ‘*a*’ and 0.8015 for the ‘*x*’. The performance comparison of the optimized model is presented in Section 5.

## 3. Numerical and Experimental Validation

In this section, the verifications of the blade design method, the numerical method, and the experimental method are all conducted based on the optimized CRF of *a* = 0.4025 and *x* = 0.8015. The verifications are conducted in the design conditions and near the stall, respectively.

### 3.1. Numerical Method

In this study, besides using the SST k-ω turbulence model to resolve the single-channel model during the optimization process to speed up the optimization process, as pointed out in the introduction, transient simulations based on the SST-DES turbulence model have been performed on the optimized blades using the CFX parallel solver to better show the flow structure and quantify the local loss.

Also mentioned in the introduction, a hybrid LES/RANS method known as the DES approach was employed for turbulence modeling. This approach was formulated based on the SST k-ω turbulence model, which includes a kinetic energy k-equation and a turbulence frequency ω-equation.

In the DES approach, LES is switched from URANS when the turbulence length predicted by URANS is larger than the local grid spacing. Therefore, the turbulence length scale in the DES approach is determined by Equation (2), while Equation (1) is used for the URANS prediction previously mentioned.

(1) $$l_{DES}=min(l_{k-\omega },\;C_{DES}∆)$$

(2) $$l_{k-\omega }=k^{0.5}/(\beta *\omega )$$

The dissipation term in the k-equation is improved as Equation (3).

(3) $$D_{DES}^{k}=\rho k^{2/3}/l_{DES}$$

**Figure 5:**
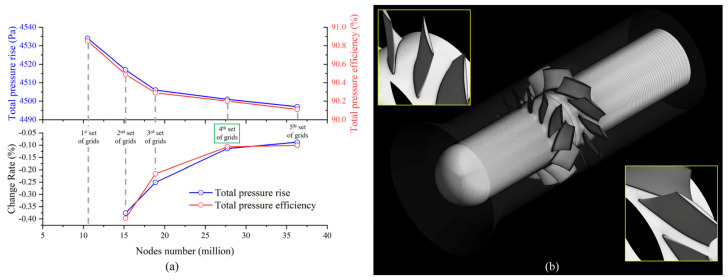
(**a**) Grid sensitivity analysis result, (**b**) the fourth set of grids layout.

For mesh generation, the control volume has been discretized via the ANSYS TURBOGRID module. After analyzing the grid sensitivity results by performing the steady-state simulation for the entire blade channel based on the SST k-ω turbulence model, as shown in Figure 5a, the fourth set of grids with 27.7 million structural grid elements was adopted, as shown in Figure 5b. y^+^ was set as 1.

For numerical discretization schemes, the advection term was calculated using a second-order advection scheme. The transient term was approximated using a second-order backward Euler scheme. The steady-state results based on the SST k-ω turbulence model were taken as the initial condition. The time-step size is 7.8227 × 10^−5^. After two rotations, the velocity and pressure field data were collected to compare with the experimental data.

### 3.2. Experimental Validation

**Figure 6:**
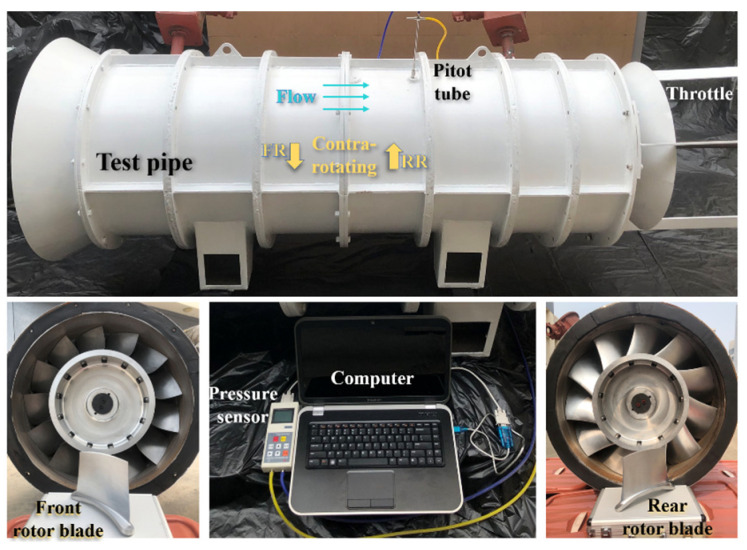
Test pipe and CRF blades.

The experimental data were gathered using a retractable pitot tube. The pressure data were transmitted to a computer via an RS232 serial communication. The range of the pressure sensor is ±8 kPa with an accuracy of ±1.0%. The test pipe with a flow throttle (used to set operating conditions) is shown in Figure 6.

**Figure 7:**
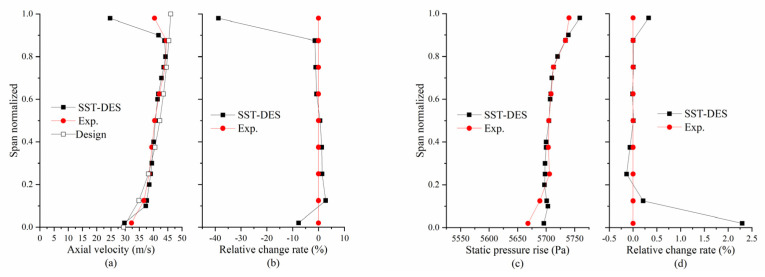
Comparison of SST-DES results against experimental data: (**a**) axial velocity, (**b**) relative change rate of the axial velocity, (**c**) static pressure rise, and (**d**) relative change rate of the static pressure rise.

To verify design and simulation accuracy, the axial velocity along the radius under design conditions is compared here, as shown in Figure 7a. Each SST-DES value in Figure 7a represents the circumference-averaged result for each corresponding span at the final time step. Each experimental value shown in Figure 7a was obtained by calculating the mean of 200 data points, which were gathered every 2 s. After analyzing the relative change rate of the simulation results compared to the experimental values, as illustrated in Figure 7b, apart from the fact that the simulated axial velocity near the annulus is smaller than the experimental results (this is mainly because when using the pitot tube, the flow field parameters near the annulus cannot be measured accurately), it can be concluded that the simulation and experimental results are in good agreement and follow a trend in line with the design expectations.

On the other hand, since the velocity fluctuation downstream of the RR is more intense when the CRF is operating near the stall, it is challenging to obtain a steady velocity value; therefore, the static pressure data along the radius were gathered, as shown in Figure 7c. After analyzing the relative change rate of the simulation results compared to the experimental values, as in Figure 7d, it can be concluded that the simulation and experimental results are in good agreement.

In summary, the flow field results of the transient simulation have been verified by the experimental results. Therefore, the following analysis is conducted based on the numerical results.

## 4. Local Entropy Production Model

Before analyzing the flow field, it is necessary to introduce the entropy production rate model used in this article and its extension under the framework of the SST-DES turbulence model.

### 4.1. Entropy Balance Equation and Entropy Production Rate (EPR) for Single-Phase Viscous Fluid

According to the first law of thermodynamics, the change in the total energy of an object over time is equal to the power of the external force plus the energy delivered to the exterior per unit time; thus, the integral energy balance equation is given by Equation (4). The left side of Equation (4) is the sum of the kinetic and internal energies of the system. The first term on the right side of Equation (4) represents the power of the volume force, the second term represents the power of the surface force, and the third term represents the conducting heat.

(4) $$\frac{D}{Dt}\underset{V}{∭}(\frac{u_{i}u_{i}}{2}+e)\rho dV=\underset{V}{∭}u_{i}k_{i}\rho dV+\iint_{S}u_{i}t_{i}dS-\iint_{S}q_{i}n_{i}dS$$

Using the Gaussian divergence theorem, the surface integral of the surface force vector field can be converted into the volume integral of its divergence. After rearrangement, the energy balance equation in the differential form can be obtained using Equation (5).

(5) $$\rho u_{i}\frac{Du_{i}}{Dt}+\rho \frac{De}{Dt}=\rho k_{i}u_{i}+u_{i}\frac{\partial \tau_{ji}}{\partial x_{j}}+\tau_{ji}\frac{\partial u_{i}}{\partial x_{j}}-\frac{\partial q_{i}}{\partial x_{i}}$$

The surface stress tensor can be decomposed into Equation (6).

(6) $$\tau_{ji}=-p\delta_{ij}+P_{ij}\;P_{ji}=\mu \left(\frac{\partial u_{i}}{\partial x_{j}}+\frac{\partial u_{j}}{\partial x_{i}}\right)-\frac{2}{3}\mu \delta_{ij}\frac{\partial u_{i}}{\partial x_{i}}$$

The appropriate term within the energy balance equation can be substituted with the definition of enthalpy, Equation (7), and the differential form of momentum conservation, Equation (8). As a result, Equation (9) can be obtained to express the evolution of the internal energy of the microfluid over time.

(7) $$h=e+\frac{p}{\rho }$$

(8) $$\rho \frac{Du_{i}}{Dt}=\rho k_{i}+\frac{\partial \tau_{ji}}{\partial x_{j}}$$

(9) $$\frac{De}{Dt}=\frac{\tau_{ji}}{\rho }\frac{\partial u_{i}}{\partial x_{j}}-\frac{1}{\rho }\frac{\partial q_{i}}{\partial x_{i}}$$

According to the first law of thermodynamics, the first term on the right side of Equation (9) represents the power of the surface stress per unit mass and the second term represents the heat supplied by the unit mass.

Depending on the decomposition of the surface stress tensor, the first term on the right side of Equation (9) can be divided into reversible and irreversible parts, as shown in Equation (10). During microfluid deformation, the presence of dynamic viscosity results in irreversibility, and the external shear stress is converted into heat. This irreversible part can be marked as *Φ*.

(10) $$\frac{\tau_{ji}}{\rho }\frac{\partial u_{i}}{\partial x_{j}}=-\frac{p}{\rho }\frac{\partial u_{i}}{\partial x_{i}}+\frac{1}{\rho }P_{ji}\frac{\partial u_{i}}{\partial x_{j}}=-\frac{p}{\rho }\frac{\partial u_{i}}{\partial x_{i}}+\frac{\Phi }{\rho }$$

In addition, the first term on the right side of Equation (10) can be transformed by the mass conservation, Equation (11), to become Equation (12).

(11) $$\frac{D\rho }{Dt}+\rho \frac{\partial u_{i}}{\partial x_{i}}=0$$

(12) $$-\frac{p}{\rho }\frac{\partial u_{i}}{\partial x_{i}}=\frac{p}{\rho^{2}}\frac{D\rho }{Dt}=-p\frac{Dv}{Dt}$$

Consequently, the external stress power of the unit mass expressed by the first term on the right-hand side of Equation (9) can be rewritten as Equation (13).

(13) $$\frac{\tau_{ji}}{\rho }\frac{\partial u_{i}}{\partial x_{j}}=-p\frac{Dv}{Dt}+\frac{\Phi }{\rho }$$

Equation (14) can be derived from the quotient rule. Bring Equation (9), Equation (13), and Equation (14) into the Gibbs energy relation, Equation (15). As a result, the entropy balance equation, Equation (16), can be obtained. The first two terms of Equation (16) characterize the rate of local entropy generation due to friction and heat conduction processes, respectively.

(14) $$\frac{\partial (q_{i}/T)}{\partial x_{i}}=\frac{1}{T}\frac{\partial q_{i}}{\partial x_{i}}-\frac{q_{i}}{T^{2}}\frac{\partial T}{\partial x_{i}}$$

(15) $$T\frac{Ds}{Dt}=\frac{De}{Dt}+p\frac{Dv}{Dt}$$

(16) $$\rho \frac{Ds}{Dt}=\frac{\Phi }{T}-\frac{q_{i}}{T^{2}}\frac{\partial T}{\partial x_{i}}-\frac{\partial (q_{i}/T)}{\partial x_{i}}$$

### 4.2. Local Entropy Production Model under SST-DES Turbulence Model

Based on the entropy balance equation and Reynolds decomposition [32], $u_{i}={\bar{u}}_{i}+u_{i}^{\prime }$, the local entropy production model under the RANS model was developed by Kock and Herwig [8], as shown in Equation (17).

(17) $$EPR=\frac{\Phi }{T}+\frac{\Phi_{\theta }}{T^{2}}=S_{PRO,\bar{D}}+S_{PRO,D^{\prime }}+S_{PRO,\bar{C}}+S_{PRO,C^{\prime }}$$

This model consists of four terms, the first two of which can be derived from the first term of Equation (16), and the last two terms from the second term of Equation (16). To apply this model to the SST-DES turbulence model, a local entropy production model for compressible media can be developed using Equations (18)–(21) to fit different turbulence length scales in the RANS or LES region.

(18) $$\begin{array}{c} S_{PRO,\bar{D}}=\frac{\mu }{T}\{\frac{2}{3}[{\left(\frac{\partial \bar{u}}{\partial x}-\frac{\partial \bar{v}}{\partial y}\right)}^{2}+{\left(\frac{\partial \bar{v}}{\partial y}-\frac{\partial \bar{w}}{\partial z}\right)}^{2}+{\left(\frac{\partial \bar{w}}{\partial z}-\frac{\partial \bar{u}}{\partial x}\right)}^{2}]+{\left(\frac{\partial \bar{v}}{\partial x}+\frac{\partial \bar{u}}{\partial y}\right)}^{2}\\ +{\left(\frac{\partial \bar{w}}{\partial y}+\frac{\partial \bar{v}}{\partial z}\right)}^{2}+{\left(\frac{\partial \bar{u}}{\partial z}+\frac{\partial \bar{w}}{\partial x}\right)}^{2}\} \end{array}$$

(19) $$S_{PRO,D^{\prime }}=\frac{\rho \beta^{*}k\omega F_{DES}}{T}\;F_{DES}=max(\frac{k^{1/2}}{\beta^{*}\omega C_{DES}∆},1)$$

(20) $$S_{PRO,\bar{C}}=\frac{\lambda }{T^{2}}[{\left(\frac{\partial T}{\partial x}\right)}^{2}+{\left(\frac{\partial T}{\partial y}\right)}^{2}+{\left(\frac{\partial T}{\partial z}\right)}^{2}]$$

(21) $$S_{PRO,C^{\prime }}=\frac{\mu_{t}}{\mu }\frac{Pr}{{Pr}_{t}}S_{PRO,\bar{C}}$$

## 5. Results

In this section, the intuitive change in airfoil shape before and after optimization, and the change in overall performance caused by the change in geometric parameters are first described.

Then, in order to determine the source of the variation firstly, several intervals have been divided near the blade along the radius. After that, the EPR was integrated within each interval to quantify their contribution to the whole blade channel before and after optimization, both in the design conditions and near stall. This step is used to locate four high-EPR regions. Next, in these four high-EPR regions, EPR distributions near the blade surface along the streamwise direction are compared.

In the last two subsections, the flow structures near a stall are analyzed from different perspectives to reveal the mechanism of local entropy production.

### 5.1. Optimized Blade Profiles and Overall Performance of CRFs

**Figure 8:**
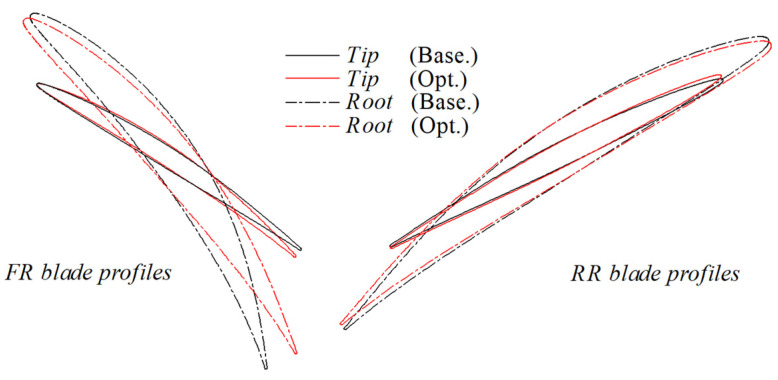
The comparison of blade profiles after optimization.

The most significant geometric changes of the blades (around the blade tip and root) after optimization were concluded in Figure 8 and Table 2 (Base. is the abbreviation for baseline, and Opt. is the abbreviation for Optimized). Around the blade tip, the value of the blade camber increased in FR and decreased in RR. Around the blade root, the value of the blade camber decreased in both FR and RR. In both the FR and RR, the incidence angle decreased around the blade tip while it increased around the root.

**Figure 9:**
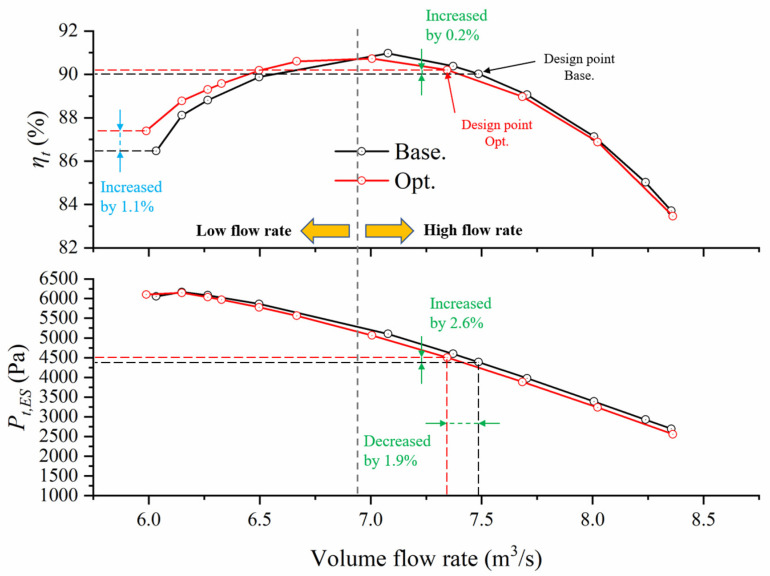
The comparison of overall performance.

As a result, in Figure 9, the design point was slightly moved to the upper left (the specific method to determine the design point has been detailed in [24]). For convenience, here, the high-flow-rate and the low-flow-rate areas were divided according to the intersection point of two efficiency curves. It can be seen that the improved degree of efficiency in the low-flow-rate area is slightly greater than the reduced degree in the high-flow-rate area. In particular, the efficiency significantly improved by approximately 1.1% near the stall, thus, expanding the stall margin and high-efficiency operating range. On the other hand, although the total pressure rises of optimized blades decreased slightly in most conditions, it increased a little bit near the stall.

In the following subsection, to reveal the aerodynamic loss mechanism, a quantitative analysis is conducted from the perspective of local aerodynamic loss generation, namely, the entropy production rate (EPR).

### 5.2. EPR at Intervals along the Radius

**Figure 10:**
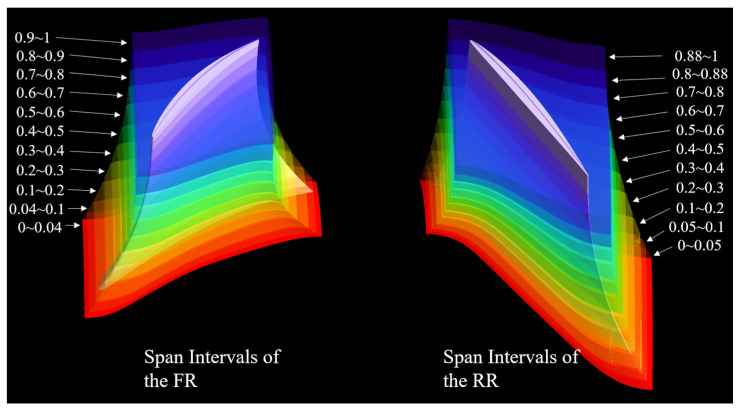
Span Intervals of the FR and RR along the radius.

As mentioned at the beginning of this section, the control volumes of the FR and RR were divided into several intervals along the radius, as shown in Figure 10. Volume integration was performed on the EPR within each interval, and the entropy production contributions of each interval were compared.

**Figure 11:**
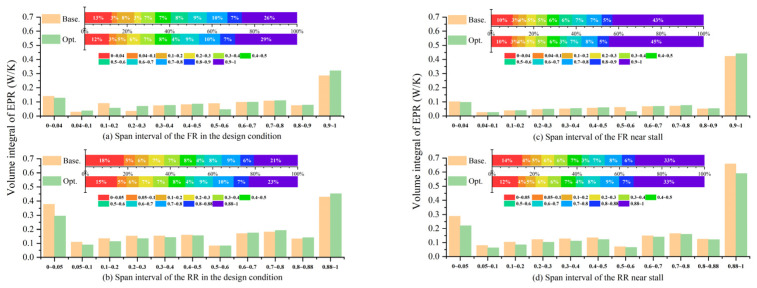
EPR contribution from each interval (along the radius) to (**a**) FRs and (**b**) RRs in the design condition, (**c**) FRs, and (**d**) RRs near the stall.

Comparing the overall trend in Figure 11a–d, respectively, regardless of whether the blades are operating in the design condition or near stall, the EPR of the RR is higher than that of the FR. Comparing Figure 11a with Figure 11c, and Figure 11b with Figure 11d, respectively, it can be observed that approaching the stall, the EPR at the topmost interval increases, while the EPR in the rest of the intervals decreases, which is a manifestation of the compensation phenomenon along the radial direction.

In Figure 11c, after optimization, when the CRF is operating close to the stall, the EPR of the FR increases, except for the intervals from 0 to 0.04 and 0.5 to 0.6; thus, the EPR of the FR manifests an overall increase, as listed in Table 3. However, as shown in Figure 11d, the EPR of the entire interval in the RR is significantly reduced, as listed in Table 3. The EPR of the entire stage is significantly reduced by 7.9% after optimization.

In Figure 11a, after optimization within the design condition—except for the significant decrease in EPR in intervals of the FR from 0 to 0.04, 0.1 to 0.2, and 0.5 to 0.6—the EPR in the rest of the intervals has been increased. Thus, the EPR of the FR manifests an overall increase, as listed in Table 3. In Figure 11b, except for a slight increase in the EPR above 0.6 spans, the EPR of the remaining intervals in the RR decreases; consequently, the EPR of the entire RR decreases. The EPR of the entire stage is reduced by 3.4% after optimization.

The contributions of each interval to the EPR in each rotor are also compared here. In Figure 11a to Figure 11d, regardless of whether the blades are operating under the design condition or near stall, the region with the highest contribution to the EPR of all the stages is around the blade tip, followed by the blade root. With respect to the loss ratio of each rotor resulting from the blade tip, it is higher in the FR than in the RR. Although the absolute value of the volume integral of the EPR of the FR is smaller than that of the RR.

After optimization, regardless of whether the blades are operating in the design condition or near stall, the EPR proportion of the blade root decreases, whereas the proportion of the blade tip increases, as shown in Figure 11a to Figure 11d. This is due to the fact that after optimization, around the blade tip, the FR’s load is added while the RR’s load is reduced, as illustrated in Figure 2. As a result, based on the assumption that the circumferential velocity components are constant at each radius before and after optimization (because the rotation speeds of both rotors are constant), the axial velocity component around the blade tip is increased compared to that at the mid-span, after the optimization, as shown in Figure 7a. However, around the blade root, the FR load decreases, whereas the RR load increases. As a result, the axial velocity component around the root area is decreased compared to that at the mid-span. A higher flow rate could lead to a more severe shear strain between the main flow and the vortices, which would increase the EPR proportion of the corresponding interval in a rotor. However, because the absolute value of the generated loss is reduced in the entire stage, especially since the loss of the RR is reduced more significantly after the optimization, thus, the efficiency of the optimized CRF is improved.

Next, several ERP contour maps along the blade passages are displayed to observe the EPR distribution phenomenon near the stall and to further localize and analyze the development of the EPR.

### 5.3. EPR Distribution along the Blade Passages

**Figure 12:**
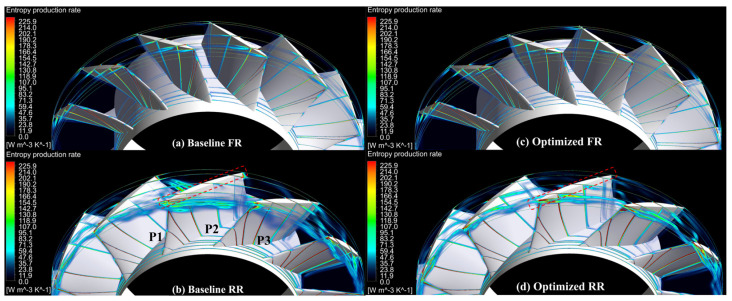
EPR distribution of the CRFs while operating near stall, (**a**) the baseline FR (**b**) the baseline RR, (**c**) the optimized FR, and (**d**) the optimized RR.

Comparing Figure 12a with Figure 12c, and Figure 12b with Figure 12d, respectively, it can be observed that after optimization, the high-EPR regions of the FR changed inconspicuously, whereas those of the RR are more obvious.

Before optimization, as shown in Figure 12b, the high-EPR regions at the blade tip grow upstream slightly in the three blade passages. These high-EPR regions are thickened and extended to the mid-span while propagating downstream. After optimization, although the high-EPR regions of the RR are still thicker than those of the FR, the stall phenomenon is effectively suppressed, as shown in Figure 12d compared to Figure 12b.

### 5.4. EPR Distribution around the Annulus

Based on the results from the last subsection, it is obvious that the concentrated region of high EPR is around the annulus; therefore, the 0.98 and 0.02 blade spans are selected to analyze the EPR distribution along the streamwise direction in more detail.

**Figure 13:**
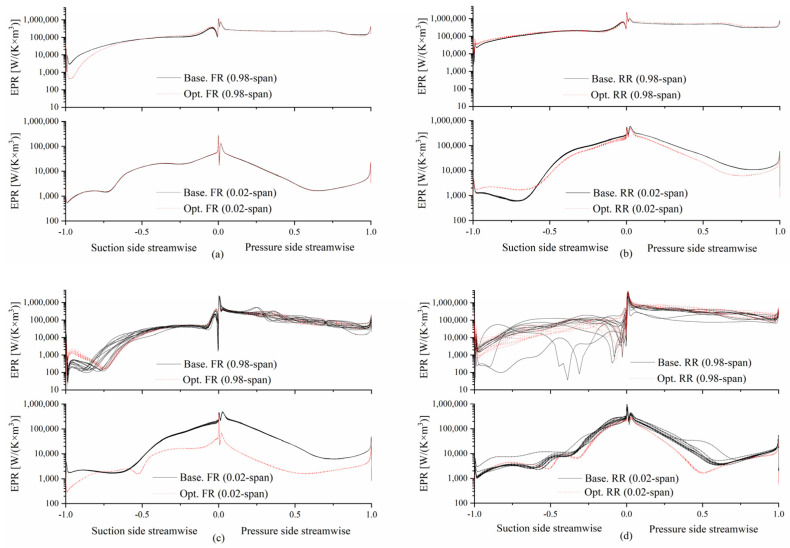
EPR distribution at (**a**) the FR, and (**b**) the RR with rotors operating in the design condition. EPR distribution at (**c**) the FR, and (**d**) the RR with rotors operating near the stall.

Figure 13 shows the EPR distribution for each blade in different rotors, respectively. From the y-coordinate scale, it can be concluded that the sequences of the four regions ranked by EPR from low to high are FR root, RR root, FR tip, and RR tip. This is marked as Sequence 1 (Figure 13c to Figure 13d to Figure 13a to Figure 13b) for ease of the subsequent description.

In these four regions, except for the highest EPR value at the leading edge (LE), the minor high value is located around 0.01 streamwise. There is also an amplitude at the trailing edge (TE) in each figure.

Comparing Figure 13a,b with Figure 13c,d, when the CRFs are operating near the stall, the fluctuation gradually increases in the order of Sequence 1, which means that the difference in the status of each blade in the rotor increases, especially in the RR tip shown in Figure 13d, echoing Figure 12b, since the high-EPR region near the LE is thickened and developed downstream only in several blade passages.

After optimization, the difference in each blade’s status in the rotor decreased, as shown in Figure 13c,d, proving that the stall has been suppressed.

### 5.5. Flow Patterns near Stall

**Figure 14:**
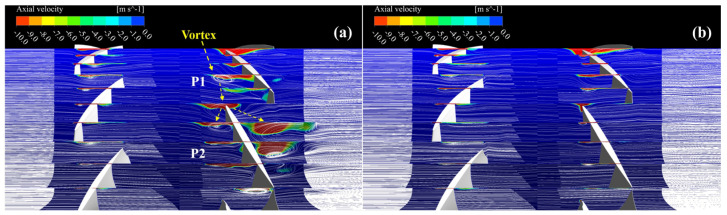
Flow pattern in several planes in (**a**) the baseline CRF, and (**b**) the optimized CRF.

To explain the above result of local entropy generation, this subsection compares and analyzes the development of the flow structure in several planes along the flow direction before and after optimization when the CRFs are operating near the stall. In Figure 14, ten planes are established within the two blade channels, P1 and P2. All of these planes pass through the rotation axis. This includes one plane located not far from the leading edge of each blade, and four additional planes that are distributed at equal angles for each blade.

In Figure 14a, in the P1 and P2, the vortex generated in front of the suction side of the RR is enlarged and develops upstream, resulting in a large recirculation region with negative-velocity components, which reduces the flow capacity near the tip of the RR. This vortex develops in the opposite direction of blade rotation, gradually weakening its intensity during flowing to the LE of the adjacent blade, and is activated again near this LE. Later, it is cut into two parts. One part is mixed in the vortex in front of the suction side of the adjacent blade, and the other part grows along the pressure side of the adjacent blade, extending its scope. After optimization, the influence range of the main tip clearance vortex structure in front of the suction surface is greatly reduced, and the interaction of the flow between blade channels is suppressed from the source. It should be noted that the dark blue color in Figure 14 shows areas of positive velocity, which means that the velocity of the fluids is not entirely zero. The upper boundary of the axial velocity contour is specifically set to zero for ease of analysis of the negative axial velocity area more clearly.

Then, the streamlines and pressure contours at 0.98 blade span are investigated to analyze the flow structure mentioned above from another perspective.

**Figure 15:**
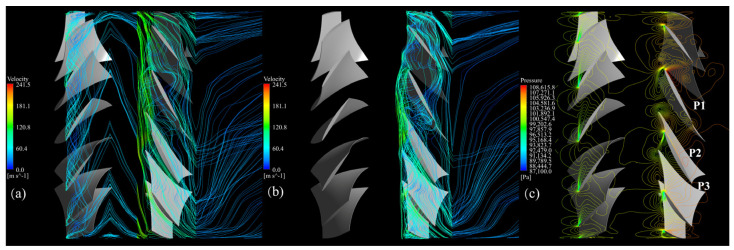
Flow pattern of the baseline CRF at the 0.98 blade span while operating near stall, (**a**) tip leakage flow of the FR, (**b**) tip leakage flow of the RR, (**c**) pressure contour of the FR and RR.

**Figure 16:**
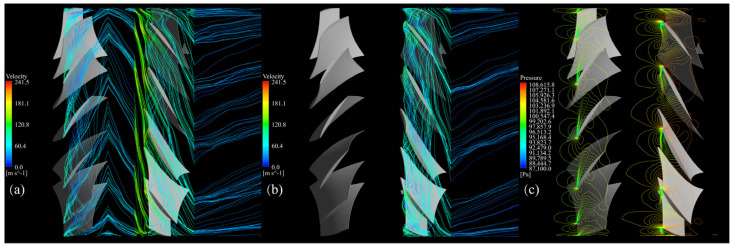
Flow pattern of the optimized CRF at the 0.98 blade span while operating near stall: (**a**) tip leakage flow of the FR, (**b**) tip leakage flow of the RR, (**c**) pressure contour of the FR and RR.

Figure 15a shows that before optimization, the tip leakage flows of the FR are developed into a chaotic flow pattern compared to that exhibited in Figure 16a in the rear stage passages. In the meanwhile, the pressure contour near the LE in the three blade passages of the RR in Figure 15c indicates that the pressure equilibrium illustrated in Figure 16c—which is required for the tip vortex structure—is fractured, leaving a chaotic pressure gradient. Thus, the structure of the RR tip leakage flow cannot be maintained in several blade passages. Furthermore, the secondary flows of the FR and RR are mixed into a more complex flow, resulting in inverted flows across a broad range. The pressure fluctuation of the rear stage also affects the pressure distribution of the front stage but does not reach the magnitude that could break the pressure equilibrium required for the tip leakage flow structure; therefore, the tip leakage flow of the FR is less affected.

### 5.6. Mechanism of EPR around the Blade Tip

According to the above brief analysis of the flow structure, this subsection will reveal the EPR mechanism around the blade tip more clearly combined with the EPR model.

**Figure 17:**
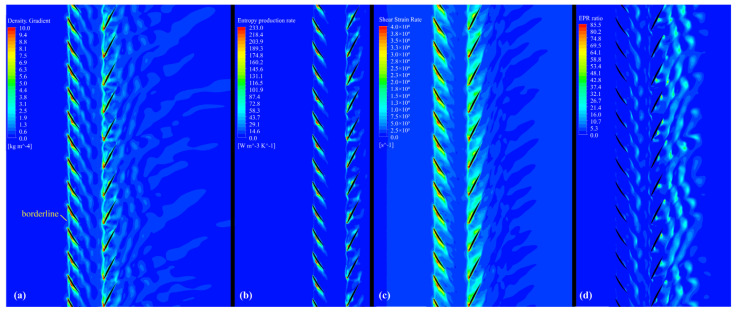
Contours at 0.98 blade span of the optimized CRF while operating near stall: (**a**) density gradient contour, (**b**) EPR contour, (**c**) shear strain rate contour, (**d**) EPR ratio contour.

**Figure 18:**
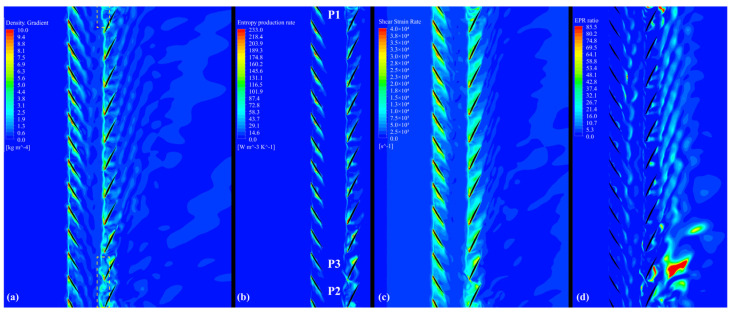
Contours at 0.98 blade span of the baseline CRF while operating near stall: (**a**) density gradient contour, (**b**) EPR contour, (**c**) shear strain rate contour, (**d**) EPR ratio contour.

According to the density gradient contour in Figure 17a, it can be observed that an apparent high-density gradient borderline is formed by the boundary of the tip leakage flow. This kind of borderline of the RR cut off the weak wake disturbance from the FR. Before optimization, in the area of the yellow rectangles in Figure 18a, the borderline of the RR is destroyed owing to the loss of balance in pressure, and it oscillates upstream, echoing the flow structure in Figure 12b, Figure 14a and Figure 15.

Comparing Figure 17b with Figure 17a, the large density gradient region basically reflects an area with a high EPR. The higher density gradient is also associated with the violent alteration of the flow structure simultaneously, as shown in Figure 17c. The region with the high-density gradient lies at the blade’s LE, wake, and boundary of the tip leakage flow. Strong shear deformations occurred at the interface between these flow structures and the main flow, resulting in irreversible loss production in CRF blade channels.

After optimization, the pressure balance required for the tip leakage flow near the stall is promised by an appropriate radial load distribution resulting from a proper design of radial load coefficient and axial spacing; therefore, the normal leakage flow structure could be maintained. Meanwhile, the flow around the blade tip is accelerated, as illustrated in Figure 7a. Combined with these two factors, the development of a low-energy fluid in front of the RR blades’ suction surface is limited, as is the expansion of the high-EPR region, which reduces the total amount of local entropy generated by the RR.

On the other hand, in the low-EPR region, the structure evolution of irreversible losses is revealed by the EPR ratio, as illustrated in Figure 17d.

(22) $$EPR\;ratio=\Phi_{\theta }/\left(T\Phi \right)=\frac{S_{PRO,\bar{C}}+S_{PRO,C^{\prime }}}{S_{PRO,\bar{D}}+S_{PRO,D^{\prime }}}$$

As these blade passages do not have thermal exchange with the exterior, and the highest Mach number is approximately 0.4, the temperature change is small throughout the passages. By comparing Figure 17d with Figure 17b, it can be observed that in the low-EPR region, heat transfer caused by viscous dissipation plays a dominant role in the EPR. The irregular flow caused by the stall phenomenon from the RR blade tip aggravates the heat transfer effect at these blade wakes in P1, P2, and P3, as shown in Figure 18d.

## 6. Conclusions

In this study, the local entropy production of a CRF is investigated by optimizing the load along the radius and conducting a flowfield analysis using an EPR model adapted to the SST-DES model. The results can be summarized as follows:

(1) The high-EPR region is found not only on the blade surface and annulus but also at the leading edge (LE), on both sides of the tip leakage flow, and around the blade wake; additionally, there is a significant density gradient in these areas. The LE of the rear rotor blade tip is identified as the position with the highest EPR across the entire blade passages;
(2) When considering low Mach number flows in a CRF without heat exchange with the exterior, the shear strain rate is the primary factor contributing to the high-EPR region, while heat transfer is the main factor contributing to the low-EPR region;
(3) The stall in the baseline model started at the leading edge (LE) of the RR blade tip, as the pressure equilibrium required for the tip leakage flow was disrupted in multiple blade passages. Additionally, the compensation phenomenon was observed along the radius in terms of the EPR distribution, when compared to the design condition;
(4) The secondary flows from the front and rear rotor blade tips combine to form a more complex flow. This leads to an enlargement of the induced secondary vortex structure and the corresponding recirculation region. At the interface between these flow structures, there is significant shear deformation, resulting in a high-EPR phenomenon;
(5) The optimization of load distribution along the radius indicates that it is necessary to restrict the accumulation of low-energy fluid near the tip leakage flow of the RR in order to maintain pressure equilibrium. Therefore, future research will focus on investigating the impact of geometric parameters of the FR and RR blade tips on the flow structure. This will help enhance the stall margin of this series of CRFs through shape optimization.

## Figures and Tables

**Table 1:** Details of the genetic algorithm.

Characteristics	Value
Population size	100
Generations	100
Elite count	10
Crossover rate	0.4
Mutation rate	adaptive

**Table 2:** Blade profile parameters.

Angles (Degree)	Tip (Base.)	Tip (Opt.)	Root (Base.)	Root (Opt.)
FR blade camber	12.22	20.58	43.91	28.97
RR blade camber	8.58	5.99	22.57	22.08
FR blade incidence	2.17	−0.24	−2.61	−0.67
RR blade incidence	2.92	−0.55	−0.50	3.88

**Table 3:** Volume integral of entropy production rate (W/K) for each rotor.

Operation	Near Stall			Design Condition		
Location	Entire	FR	RR	Entire	FR	RR
Base.	3.004	0.985	2.018	3.177	1.098	2.079
Opt.	2.768	0.990	1.778	3.070	1.100	1.970

## Data Availability

The data are not publicly available due to privacy.

## Abbreviations

Roman Letters	
*a*	load parameter
*C_DES_*	constant in the blending function for the DES model, *C_DES_* = 0.61
*C_u_*	the tangential component of absolute velocity [m/s]
*∆C_u_*	increment of the tangential component of absolute velocity [m/s]
*C_z_*	the axial component of absolute velocity [m/s]
*e*	specific internal energy [m^2^/s^2^]
*h*	specific enthalpy [m^2^/s^2^]
*i_opt_*	optimal incidence angle [degree]
*k*	turbulence kinetic energy [m^2^/s^2^]
*k_i_*	volume force vector [N]
*l_FR_*	length of the chamber line at the mid-span of the FR [m]
*l_RR_*	length of the chamber line at the mid-span of the RR [m]
*n_i_*	unit normal vector to a surface
*P_ji_*	surface stress tensor without normal stress component [N/m^2^]
*P_t,in_*	averaged total pressure rise at the inlet [Pa]
*P_t,ES_*	total pressure rise of the entire stage [Pa]
*P_t,FR_*	total pressure rise of the FR [Pa]
*P_t,RR_*	total pressure rise of the RR [Pa]
*P_t,out_*	averaged total pressure rise at the outlet [Pa]
*Pr*	Prandtl number
*Pr_t_*	turbulent Prandtl number
*P1*	passage 1
*P2*	passage 2
*P3*	passage 3
*p*	pressure [Pa]
*q_i_*	heat flux [W/m^2^]
*r*	radius [m]
$S_{PRO,\bar{C}}$	entropy production rate by heat transfer with mean temperature gradients [W/(K·m^3^)]
$S_{PRO,C^{\prime }}$	entropy production rate by heat transfer with fluctuating temperature gradients [W/(K·m^3^)]
$S_{PRO,\bar{D}}$	entropy production rate by direct dissipation [W/(K·m^3^)]
$S_{PRO,D^{\prime }}$	entropy production rate by turbulent dissipation [W/(K·m^3^)]
*s*	entropy [J/K]
*T*	temperature [K]
*t*	time [s]
*t_i_*	surface force vector [N]
*u_i_*	velocity vector [m/s]
${\bar{u}}_{i}$	steady component of velocity vector [m/s]
$u_{i}^{\prime }$	fluctuation of velocity vector [m/s]
*v*	specific volume [m^3^/kg]
*W*	relative velocity [m/s]
*x*	axial spacing parameter
*x_i_*	displacement vector [m]
Greek Letters	
*β**	constant in the k-ω model, *β** = 0.09
*δ_ij_*	Kronecker delta
*∆*	grid spacing [m]
*η_t_*	total pressure efficiency of the CRF [%]
*λ*	thermal conductivity [W/(m·K)]
*μ*	dynamic viscosity [Pa·s]
*μ_t_*	eddy viscosity [Pa·s]
*ρ*	density [kg/m^3^]
*τ_ji_*	surface stress tensor [N/m^2^]
*Φ*	viscous dissipation of mechanical energy [W/m^3^]
$\Phi_{\theta }/T^{2}$	EPR by heat transfer [W/(K·m^3^)]
*ω*	turbulence eddy frequency [1/s]
